# Comparative evaluation of the sarcopenia index and 5 related creatinine–cystatin C biomarkers in relation to all-cause mortality in older adults: A cohort study

**DOI:** 10.1097/MD.0000000000050842

**Published:** 2026-10-02

**Authors:** Ya Liu, Yingsong Qi, Huanhuan Wang, Jin Zhou, Xiaojing Zhao, Min Zhang, Ce Liu, Jie Shen, Shuzhong Duan

**Affiliations:** a Department of Nephrology, Affiliated Hospital of Chengde Medical University, Chengde, Hebei, China; b Department of Cardiothoracic Surgery, Chengde Central Hospital, Chengde, Hebei, China; c Department of Nephrology, Xiangtan City Central Hospital, Xiangtan, Hunan, China; d Department of Rehabilitation Medicine, Chengde Central Hospital, Chengde, Hebei, China.

**Keywords:** all-cause mortality, comparative biomarker study, creatinine–cystatin C biomarkers, older adults, prognostic biomarkers, sarcopenia index

## Abstract

Several creatinine- and cystatin C-based biomarkers have been individually associated with mortality, but their comparative associations have rarely been evaluated head-to-head under a common analytic framework. We compared 6 related biomarkers in relation to all-cause mortality in older adults. In the China Health and Retirement Longitudinal Study 2015 to 2020 cohort, we studied 2987 men (428 deaths) and 2995 women (228 deaths) aged ≥60 years. Six related biomarkers (sarcopenia index [SI], predicted skeletal muscle index, total body muscle mass, creatinine-to-cystatin C ratio [CCR], eGFRcys(2012)/eGFRcr(2021), and eGFRcys(2012) − eGFRcr(2021)) were evaluated using sex-stratified Cox models, a prespecified mutually adjusted bootstrap ranking framework, and supportive external evidence from the National Health and Nutrition Examination Survey. In fully adjusted single-biomarker models, SI, predicted skeletal muscle index, and total body muscle mass showed the most consistent inverse associations with all-cause mortality, whereas CCR, eGFRcys(2012)/eGFRcr(2021), and eGFRcys(2012) − eGFRcr(2021) were weaker, particularly in women. Within the joint 6-biomarker framework, SI ranked highest in both sexes, whereas CCR showed a reversed association after mutual adjustment. Discrimination was broadly similar across single biomarkers, and the joint 6-biomarker model provided only modest improvement beyond standard covariates. Supportive analyses, including the National Health and Nutrition Examination Survey, were directionally consistent overall but less precise. Among 6 closely related creatinine–cystatin C biomarkers, SI showed the most consistent comparative association pattern with all-cause mortality. However, incremental discrimination beyond standard covariates was small, supporting SI primarily as the clearest comparative marker within this biomarker family rather than as evidence of major predictive superiority.

## 1. Introduction

Sarcopenia is a major contributor to disability, loss of independence, and premature mortality in older adults.^[1–4]^ Because muscle-related vulnerability is at least partially modifiable, earlier recognition may facilitate closer monitoring and the targeting of supportive interventions.^[5]^ However, direct assessment of muscle mass and physical performance is often impractical in routine geriatric practice, particularly in large-scale cohort studies and resource-constrained settings. This limitation has increased interest in laboratory-derived markers that may help identify older adults at higher risk.^[6,7]^

Creatinine and cystatin C are widely used renal biomarkers in clinical practice, but they differ in non-glomerular filtration rate (non-GFR) determinants that are particularly relevant to aging. Creatinine is strongly influenced by muscle mass and creatinine generation, whereas cystatin C is less muscle-dependent than creatinine and may better preserve filtration-related information when muscle mass is atypical.^[8–12]^ These measures should therefore not be viewed as interchangeable filtration markers, because their risk relationships may vary according to body composition and, potentially, by sex.^[8–14]^ Their combination has generated a family of related creatinine–cystatin C-based biomarkers, including creatinine filtration-type constructs, ratio-based indices, discordance measures, and anthropometrically informed composites, each of which may capture overlapping but differently weighted filtration-related and non-GFR information.^[13,15–17]^ In the present study, we use the term sarcopenia index (SI) specifically to denote serum creatinine × cystatin C-based estimated glomerular filtration rate (Cr × eGFRcys), distinct from the ratio-based SI, typically defined as serum creatinine/cystatin C × 100.

Several individual creatinine–cystatin C-based biomarkers and discordance measures have been linked to mortality and adverse outcomes in both disease-specific and general-population settings.^[17–23]^ For example, creatinine filtration-type indices have been associated with frailty and mortality,^[13]^ multiple creatinine–cystatin C serum indices have predicted mortality in non-dialysis CKD,^[20]^ and eGFR discordance itself has been associated with mortality in the general population.^[21–23]^ However, because these closely related biomarkers share mathematical components and exhibit substantial collinearity, simple side-by-side single-biomarker associations are insufficient to determine which marker most consistently retains comparative prognostic information within this biomarker family. This uncertainty creates a practical interpretive problem for clinical and epidemiological research, particularly in older adults for whom direct assessment of muscle-related impairment is often unavailable. To address this gap, we used the China Health and Retirement Longitudinal Study (CHARLS), a nationally representative cohort of older Chinese adults,^[24]^ to compare 6 related creatinine–cystatin C biomarkers in relation to all-cause mortality within a prespecified comparative framework that explicitly accounted for their shared components and substantial collinearity. Because these biomarkers are highly correlated and likely reflect overlapping biological information in different proportions, our aim was not simply to identify a single universally best marker, but to determine which one showed the most consistent comparative association pattern when evaluated side by side. We therefore prioritized single-biomarker associations and a prespecified mutually adjusted bootstrap ranking framework, while treating discrimination analyses and the supportive external analysis in the National Health and Nutrition Examination Survey (NHANES) as secondary.

## 2. Methods

### 2.1. Study population

We used the 2015 baseline of the CHARLS, a nationally representative cohort of Chinese adults, and restricted analyses to participants aged ≥60 years with baseline creatinine and cystatin C measurements and available mortality follow-up.^[24]^ After prespecified exclusions, the final analytic cohort included 5982 participants (men: 2987; women: 2995; Fig. 1). All participants provided written informed consent, and the study was approved by the Peking University Institutional Review Board. Baseline characteristics of the analytic cohort are shown in Table 1, and inclusion-versus-exclusion characteristics are summarized in Table S1, Supplemental Digital Content 2. The primary outcome was all-cause mortality through 2020, identified in follow-up waves.

**Table 1:** Baseline characteristics of the CHARLS analytic cohort (2015), stratified by sex.

Characteristic	Overall N = 5982	Men N = 2987	Women N = 2995
CCR	0.93 (0.26)	1.01 (0.25)	0.84 (0.24)
pSMI	6.90 (0.94)	7.51 (0.76)	6.29 (0.66)
SI	68.0 (21.0)	77.0 (20.0)	60.0 (17.0)
eGFRratio	0.98 (0.28)	0.98 (0.26)	0.97 (0.30)
eGFRdiff, mL/min/1.73 m^2^	−4.0 (16.0)	−3.0 (16.0)	−5.0 (16.0)
TBMM	37.0 (7.0)	43.0 (6.0)	32.0 (4.0)
Age, yr	68.0 (6.0)	68.0 (6.0)	68.0 (7.0)
Residence area			
Rural village	3811 (64.0%)	1929 (65.0%)	1882 (63.0%)
Urban community	2171 (36.0%)	1058 (35.0%)	1113 (37.0%)
Height, cm	157.0 (9.0)	163.0 (7.0)	151.0 (6.0)
Weight, kg	58.0 (11.0)	61.0 (11.0)	55.0 (10.0)
Waist-to-height ratio	0.55 (0.07)	0.52 (0.06)	0.58 (0.07)
Triglycerides, mg/dL	111 (81, 163)	98 (74, 143)	124 (90, 179)
LDL cholesterol, mg/dL	104.0 (29.0)	99.0 (28.0)	109.0 (29.0)
hs-CRP, mg/L	1.50 (0.80, 2.80)	1.40 (0.80, 2.70)	1.50 (0.80, 2.80)
Smoking	1680 (28.0%)	1501 (50.0%)	179 (6.0%)
Lung disease	1009 (17.0%)	597 (20.0%)	412 (14.0%)
Alcohol drinking	2830 (47.0%)	2139 (72.0%)	691 (23.0%)
Hypertension	2520 (42.0%)	1183 (40.0%)	1337 (45.0%)
Diabetes	727 (12.0%)	303 (10.0%)	424 (14.0%)
Cancer	103 (1.7%)	41 (1.4%)	62 (2.1%)
Stroke	294 (4.9%)	167 (5.6%)	127 (4.2%)
Psychiatric disease	136 (2.3%)	48 (1.6%)	88 (2.9%)
Liver disease	394 (6.6%)	209 (7.0%)	185 (6.2%)
Kidney disease	611 (10.0%)	362 (12.0%)	249 (8.3%)
Joint disease	2833 (47.0%)	1214 (41.0%)	1619 (54.0%)
Sarcopenia (AWGS-aligned)			
No	4895 (82.0%)	2465 (83.0%)	2430 (81.0%)
Yes	1087 (18.0%)	522 (17.0%)	565 (19.0%)

Mean (SD), median (IQR), or n (%) are shown in the table.

AWGS = Asian Working Group for Sarcopenia, CCR = creatinine-to-cystatin C ratio, CHARLS = China Health and Retirement Longitudinal Study, eGFRdiff = eGFRcys(2012) − eGFRcr(2021), eGFRratio = eGFRcys(2012)/eGFRcr(2021), hs-CRP = high-sensitivity C-reactive protein, LDL = low-density lipoprotein, pSMI = predicted skeletal muscle index, SD = standard deviation, SI = sarcopenia index (Cr × eGFRcys), TBMM = total body muscle mass.

**Figure 1.**
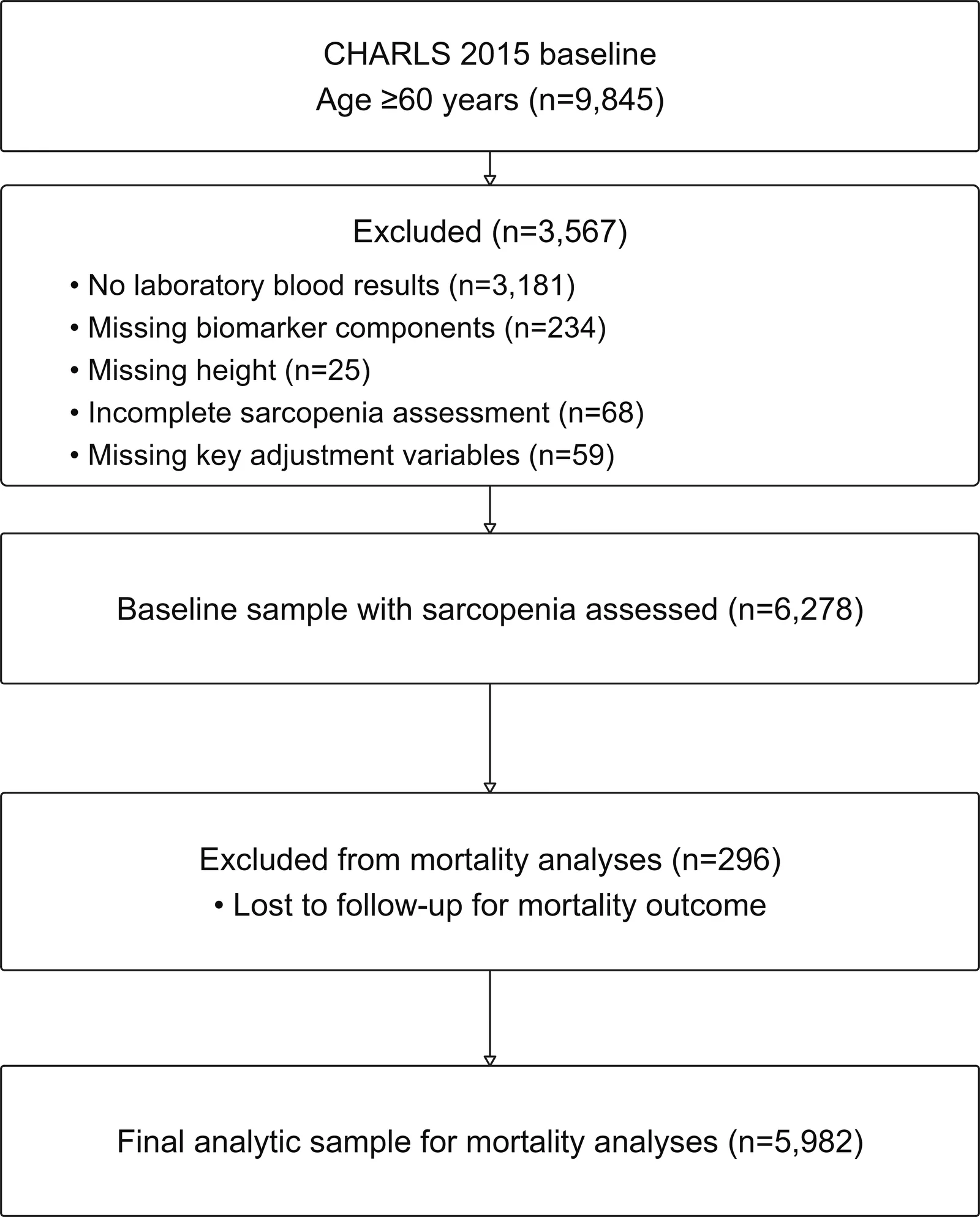
Flowchart of participant selection in CHARLS. Participants aged ≥60 years at the 2015 baseline (n = 9845); exclusions: no laboratory blood results (n = 3181), missing biomarker components (n = 234), missing height (n = 25), incomplete sarcopenia assessment (n = 68), and missing key adjustment variables (n = 59). Baseline sample with sarcopenia assessed (n = 6278); excluded due to loss to mortality follow-up (n = 296); final analytic sample (n = 5982). Exclusion categories were applied sequentially and are mutually exclusive. “No laboratory blood results” indicates absence of baseline laboratory blood testing, whereas “Missing biomarker components” indicates that laboratory testing was available but one or more required biomarker components were unavailable for calculation. CHARLS = China Health and Retirement Longitudinal Study.

### 2.2. Laboratory measurements

Serum creatinine was measured using a rate-blanked and compensated Jaffé method, whereas cystatin C was measured using a particle-enhanced turbidimetric immunoassay.^[17]^ Serum assays were conducted at certified laboratories following standardized protocols, with quality-control measures including internal control samples. The available CHARLS documentation did not provide specific details on assay calibration standards or coefficients of variation. Accordingly, whether creatinine calibration was traceable to isotope-dilution mass spectrometry or cystatin C calibration was traceable to an IFCC/ERM-DA471 reference material could not be confirmed and was therefore not assumed in the present analysis.

### 2.3. Biomarker derivation

We evaluated 6 related creatinine–cystatin C biomarkers: the creatinine-to-cystatin C ratio (CCR), SI, eGFRratio (eGFRcys(2012)/eGFRcr(2021)), eGFRdiff (eGFRcys(2012) − eGFRcr(2021)), predicted skeletal muscle index (pSMI), and total body muscle mass (TBMM). Here, SI was defined as serum creatinine multiplied by the cystatin C-based estimated glomerular filtration rate (Cr × eGFRcys), whereas the CCR was treated separately. This terminology was chosen to avoid confusion with the ratio-based “sarcopenia index” used in some previous studies. The Chronic Kidney Disease Epidemiology Collaboration 2012 cystatin C equation was used as the established cystatin C-only equation for the cystatin C-based constructs, whereas the Chronic Kidney Disease Epidemiology Collaboration 2021 creatinine equation was selected to provide a contemporary race-free creatinine-based eGFR estimate.^[25,26]^ Creatinine and cystatin C were entered in assay-reported units. Detailed definitions, component equations, input units, and references are provided in Table S2, Supplemental Digital Content 3.

To enable direct comparisons across indices, all biomarkers were standardized within sex as *z*-scores, and hazard ratios (HRs) were reported per 1-standard deviation (SD) increment.

### 2.4. Covariates

We applied a prespecified hierarchical adjustment approach. Model 1 adjusted for age and waist-to-height ratio. Model 2 additionally adjusted for residence area, smoking, drinking, lung disease, hypertension, diabetes, cancer, stroke, psychiatric disease, liver disease, kidney disease, joint disease, log triglycerides, and log high-sensitivity C-reactive protein. Model 3† was prespecified as a sensitivity model and additionally adjusted for Asian Working Group for Sarcopenia-aligned sarcopenia based on estimated muscle mass,^[27]^ handgrip strength,^[2]^ and physical performance defined using a previously published CHARLS-based implementation.^[28]^ This model was included to assess whether the comparative biomarker associations were materially altered after further accounting for baseline sarcopenia-related status, rather than to define the primary analytical model.

### 2.5. Statistical analysis

The primary objective was to compare the relative ordering of the 6 biomarkers in relation to all-cause mortality within a prespecified comparative framework. Sex-stratified Cox proportional hazards models were first fitted for each biomarker separately to estimate single-biomarker associations. A mutually adjusted joint Cox model including all 6 biomarkers was then fitted, and comparative ranking was summarized using bootstrap Best probability, defined as the proportion of bootstrap resamples in which a biomarker had the lowest HR per 1-SD increment in the joint model. Joint-model analyses were restricted to sex-specific complete-case samples defined in advance, with complete data on the outcome, all 6 biomarkers, and Model 2 covariates. Locked sample definitions for the comparative analyses and incremental discrimination comparisons are detailed in Methods S1, Supplemental Digital Content 1.

Because these biomarkers share core components and exhibit substantial multicollinearity, coefficients from the mutually adjusted model were interpreted as comparative conditional signals within a correlated biomarker family rather than as isolated mechanistic effects. Accordingly, Best probability was used as a complementary measure of comparative ranking stability across bootstrap resamples, rather than as a replacement for inference from the mutually adjusted Cox model or as evidence of biological independence of any single biomarker. In single-biomarker analyses, biomarkers were examined both as continuous standardized variables and by quartiles; natural cubic splines were used to characterize the gradient and shape of associations.

Discrimination and incremental discrimination were prespecified as secondary analyses, as performance differences were expected to be modest given the high intercorrelation among biomarkers. Additional supportive analyses included pairwise coefficient contrasts and inverse-probability weighting (IPW) for complete-case inclusion. Proportional hazards assumptions were assessed using Schoenfeld residuals.^[29]^ Where nominal deviations were observed, HRs were interpreted as time-averaged effects over follow-up. Detailed procedures for bootstrap analyses, supportive external analysis in NHANES, weighting, and software and reproducibility are provided in Methods S2 to S7, Supplemental Digital Content 1.

### 2.6. Supportive external analysis in NHANES

As a secondary exploratory analysis, we conducted a supportive external analysis using the NHANES 1999 to 2004 cystatin C subsample to assess whether the broader comparative pattern observed in CHARLS was directionally supported. We applied parallel survey-weighted, sex-stratified Cox models with covariates that closely paralleled the CHARLS Model 2 set; because public NHANES does not provide a geographic-context variable directly comparable to the CHARLS area measure, poverty-to-income ratio was used as an available proxy for socioeconomic context. Given differences in sample size, event counts, and cohort design, NHANES was used as supportive external evidence rather than as a formal validation cohort. Detailed methods, including an additional NHANES-derived appendicular skeletal muscle mass proxy calculated using the published equation from Shi et al^[30]^ and dual-energy X-ray absorptiometry (DXA)-adjusted sensitivity analyses, are provided in Methods S5, Supplemental Digital Content 1.

## 3. Results

### 3.1. Single-biomarker associations

In CHARLS, 656 deaths occurred over follow-up (428 among men and 228 among women). In fully adjusted single-biomarker Model 2 analyses, SI, pSMI, and TBMM showed the most consistent inverse associations with all-cause mortality, whereas CCR, eGFRratio, and eGFRdiff showed weaker associations, particularly in women (Table 2). For SI, the per 1-SD HR was 0.77 (95% confidence interval [CI], 0.68–0.88) in men and 0.79 (0.65–0.96) in women; corresponding estimates were 0.73 (0.64–0.85) and 0.70 (0.58–0.85) for pSMI, and 0.72 (0.63–0.82) and 0.69 (0.58–0.81) for TBMM. In men, all 6 biomarkers were inversely associated with mortality in Model 2, whereas in women the weaker signals were concentrated in CCR, eGFRratio, and eGFRdiff. Quartile analyses were directionally consistent (Table S3, Supplemental Digital Content 4), and spline analyses showed steeper risk elevation at lower biomarker values than additional protection at higher values (Fig. 2). Descriptive distributions of the 6 biomarkers according to Asian Working Group for Sarcopenia-aligned sarcopenia status, stratified by sex, are shown in Figure S1, Supplemental Digital Content 12.

**Table 2:** Sex-specific associations of 6 related creatinine–cystatin C biomarkers with all-cause mortality in CHARLS 2015 to 2020 (per 1-SD increase).

Biomarker	Men				Women			
	N = 2987, Death = 428				N = 2995, Death = 228			
	Unadjusted	Model 1	Model 2	Model 3†	Unadjusted	Model 1	Model 2	Model 3†
CCR	0.67 (0.59–0.77)***	0.78 (0.69–0.89)***	0.83 (0.74–0.94)**	0.85 (0.73–0.98)**	0.65 (0.53–0.80)***	0.82 (0.68–0.99)*	0.85 (0.71–1.02)	0.85 (0.63–1.02)
pSMI	0.63 (0.57–0.70)***	0.70 (0.61–0.81)***	0.73 (0.64–0.85)***	0.80 (0.69–0.93)**	0.55 (0.48–0.64)***	0.72 (0.60–0.86)***	0.70 (0.58–0.85)***	0.74 (0.59–0.93)**
SI	0.55 (0.48–0.62)***	0.70 (0.62–0.81)***	0.77 (0.68–0.88)***	0.79 (0.66–0.91)***	0.49 (0.40–0.59)***	0.74 (0.61–0.91)**	0.79 (0.65–0.96)*	0.79 (0.59–0.98)*
eGFRratio	0.63 (0.55–0.72)***	0.76 (0.66–0.86)***	0.81 (0.72–0.92)**	0.83 (0.69–0.95)**	0.66 (0.52–0.83)***	0.85 (0.71–1.02)	0.88 (0.74–1.05)	0.88 (0.65–1.06)
eGFRdiff	0.68 (0.61–0.75)***	0.75 (0.68–0.84)***	0.80 (0.72–0.89)***	0.82 (0.72–0.93)***	0.74 (0.64–0.85)***	0.84 (0.73–0.97)*	0.87 (0.76–1.01)	0.87 (0.74–1.01)
TBMM	0.64 (0.58–0.71)***	0.68 (0.59–0.78)***	0.72 (0.63–0.82)***	0.78 (0.66–0.90)***	0.55 (0.48–0.63)***	0.68 (0.58–0.80)***	0.69 (0.58–0.81)***	0.71 (0.57–0.88)***

Model 1 adjusted for age and waist-to-height ratio (WHtR). Model 2 additionally adjusted for residence area, smoking, drinking, lung disease, hypertension, diabetes, cancer, stroke, psychiatric disease, liver disease, kidney disease, joint disease, log triglycerides (logTG), and log C-reactive protein (logCRP).

AWGS = Asian Working Group for Sarcopenia, CCR = creatinine-to-cystatin C ratio, CHARLS = China Health and Retirement Longitudinal Study, eGFRdiff = eGFRcys(2012) − eGFRcr(2021), eGFRratio = eGFRcys(2012)/eGFRcr(2021), pSMI = predicted skeletal muscle index, SD = standard deviation, SI = sarcopenia index (Cr × eGFRcys), TBMM = total body muscle mass, WHtR = waist-to-height ratio.

† Model 3 was a prespecified sensitivity model that additionally adjusted for AWGS-aligned sarcopenia.

* *P* < .05.

** *P* < .01.

*** *P* < .001.

**Figure 2.**
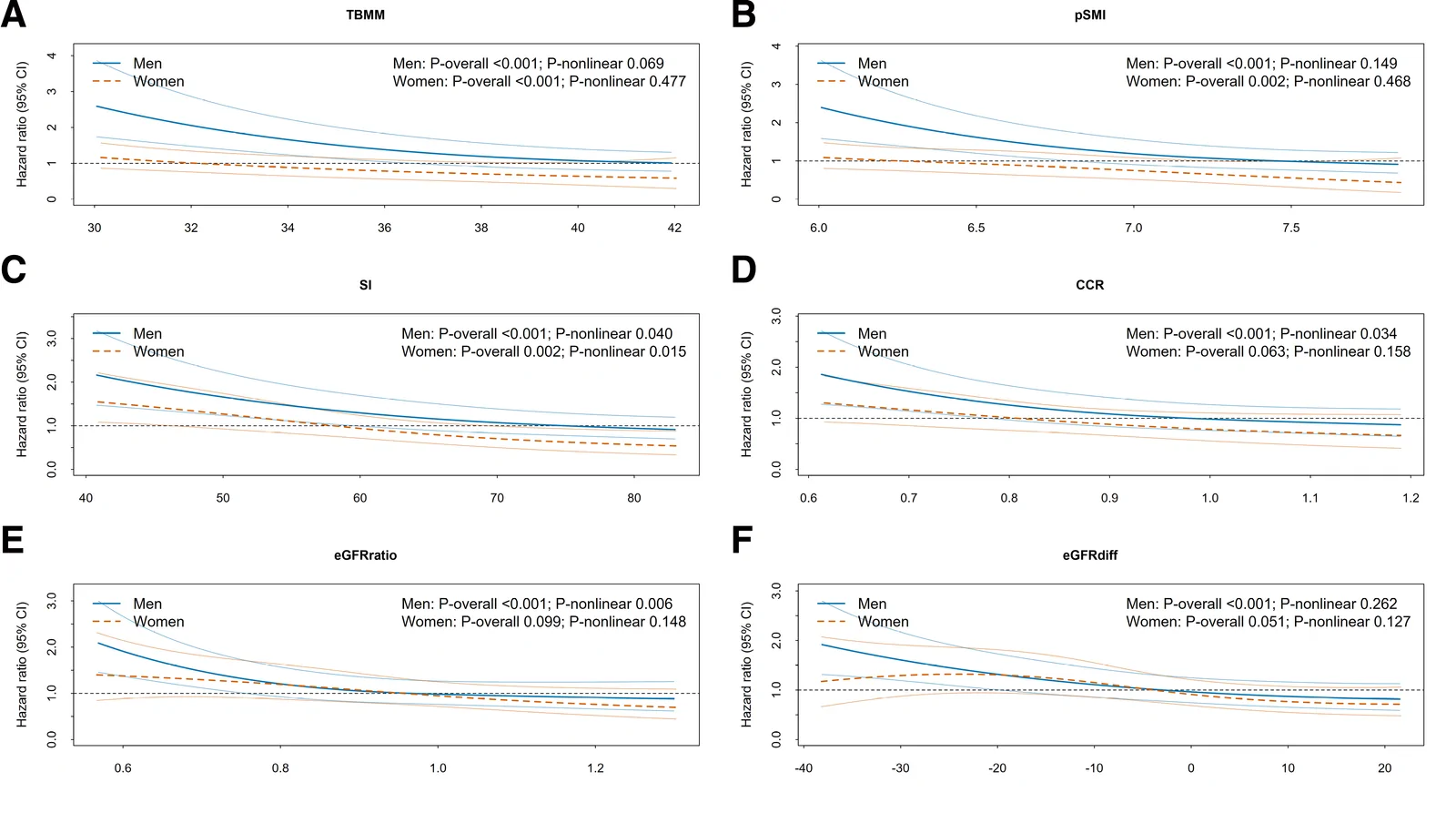
Sex-specific natural cubic spline (df = 3) associations of 6 related creatinine–cystatin C biomarkers with all-cause mortality in CHARLS. (A) TBMM; (B) pSMI; (C) SI; (D) CCR; (E) eGFRratio; and (F) eGFRdiff. Curves show hazard ratios from sex-specific single-biomarker Cox models adjusted for Model 2 covariates. Hazard ratios are plotted relative to the sex-specific median value (reference = 1.0). CCR = creatinine-to-cystatin C ratio, CHARLS = China Health and Retirement Longitudinal Study, eGFRdiff = eGFRcys(2012) − eGFRcr(2021), eGFRratio = eGFRcys(2012)/eGFRcr(2021), pSMI = predicted skeletal muscle index, SI = sarcopenia index, TBMM = total body muscle mass.

### 3.2. Joint-model bootstrap ranking

Within the prespecified, mutually adjusted, 6-biomarker Cox bootstrap framework, SI ranked highest in both sexes (Table 3). In the joint model, SI had the lowest HR and the highest Best probability (women: HR, 0.27; 95% CI, 0.13–0.56; Best probability, 94.7%; men: HR, 0.46; 95% CI, 0.30–0.71; Best probability, 87.5%). CCR showed sign inversion after mutual adjustment. Pairwise contrasts indicated that SI had a more protective joint-model coefficient than CCR and eGFRratio in both sexes (Table S4, Supplemental Digital Content 5).

**Table 3:** Mutually adjusted hazard ratios for all-cause mortality from a 6-biomarker joint model, by sex in CHARLS 2015 to 2020.

Biomarker	Men		Women	
	N = 2987, Death = 428		N = 2995, Death = 228	
	HR per SD (95% CI)	Best probability (%)	HR per SD (95% CI)	Best probability (%)
SI	0.463 (0.304–0.705)	87.5	0.272 (0.132–0.563)	94.7
pSMI	0.936 (0.547–1.601)	2.1	1.013 (0.628–1.634)	0.3
CCR	1.882 (1.328–2.666)	0.0	2.473 (1.304–4.690)	0.1
eGFRratio	1.229 (0.802–1.884)	0.0	1.439 (0.758–2.730)	0.1
TBMM	0.813 (0.482–1.374)	8.5	0.659 (0.385–1.128)	4.7
eGFRdiff	0.833 (0.624–1.112)	1.9	1.067 (0.732–1.555)	0.1

Hazard ratios (HRs) are presented per 1-SD increase (sex-specific *z*-scores). Sex-stratified Cox models included all 6 biomarkers simultaneously and were adjusted for Model 2 covariates (as defined in Methods). Best probability reports bootstrap ranking stability (*B* = 1000), defined as the probability that a biomarker had the most protective standardized coefficient (equivalently, the lowest HR) in the mutually adjusted model. In the present analyses, all biomarkers had NonNA = 1000; therefore, Best probability denominators did not differ across biomarkers. Best probability is intended as a stability-oriented comparative summary under severe collinearity rather than a direct measure of standalone clinical utility. Collinearity among the 6 biomarkers was assessed using variance inflation factor (VIF) and tolerance (Table S6, Supplemental Digital Content 7).

CCR = creatinine-to-cystatin C ratio, CHARLS = China Health and Retirement Longitudinal Study, CI = confidence interval, eGFRdiff = eGFRcys(2012) − eGFRcr(2021), eGFRratio = eGFRcys(2012)/eGFRcr(2021), NonNA = number of non-missing (finite) bootstrap estimates, pSMI = predicted skeletal muscle index, SD = standard deviation, SI = sarcopenia index (Cr × eGFRcys), TBMM = total body muscle mass.

### 3.3. Discrimination and incremental discrimination

Secondary performance analyses showed broadly similar optimism-corrected Harrell’s C-index values across single-biomarker models (Table 4). In paired in-sample comparisons against the covariates-only baseline, the joint 6-biomarker model yielded only modest incremental discrimination, with ΔC-index 0.0124 (95% CI, 0.0047–0.0245) in men and 0.0108 (0.0012–0.0284) in women; ΔAUC_5y was 0.0152 (0.0036–0.0327) in men and 0.0044 (−0.0107 to 0.0314) in women (Table S5, Supplemental Digital Content 6). Multicollinearity diagnostics for the 6-biomarker joint model are summarized in Table S6, Supplemental Digital Content 7.

**Table 4:** Optimism-corrected discrimination, 5-year calibration, and secondary incremental discrimination for sex-specific single-biomarker Model 2 Cox models in CHARLS 2015 to 2020.

Biomarker	Men					Women				
	N = 2987, Death = 428					N = 2995, Death = 228				
	Harrell C-index	Brier	CIL	CS	ΔC	Harrell C-index	Brier	CIL	CS	ΔC
CCR	0.716	0.113	0.016	0.949	0.005	0.744	0.065	0.011	0.929	0.002
pSMI	0.719	0.112	0.011	0.957	0.006	0.743	0.065	0.007	0.920	0.004
SI	0.720	0.112	0.017	0.955	0.008	0.745	0.065	0.012	0.923	0.005
eGFRratio	0.718	0.112	0.014	0.960	0.006	0.740	0.065	0.011	0.917	0.001
eGFRdiff	0.720	0.112	0.011	0.961	0.007	0.743	0.065	0.011	0.927	0.002
TBMM	0.718	0.111	0.005	0.947	0.007	0.748	0.065	0.013	0.927	0.007

Values are optimism-corrected estimates from bootstrap resampling (*B* = 200) for sex-stratified single-biomarker Model 2 Cox models in CHARLS. Biomarkers were entered as sex-specific *z*-scores (HRs per 1-SD increase). Harrell’s C-index reflects overall discrimination over follow-up, whereas Brier, CIL (calibration-in-the-large), and CS (calibration slope) summarize 5-year calibration. Secondary incremental discrimination is summarized by ΔC, defined as the difference in optimism-corrected Harrell’s C-index between the biomarker model and a covariates-only baseline. Uncertainty intervals for paired-bootstrap apparent incremental discrimination, including the 6-biomarker joint model, are provided in Table S5, Supplemental Digital Content 6. These paired-bootstrap Δ estimates quantify apparent (in-sample) gains rather than external, transportability, or outer cross-validation performance. Model 2 covariates are defined in Table 2 and Methods.

CHARLS = China Health and Retirement Longitudinal Study, eGFRdiff = eGFRcys(2012) − eGFRcr(2021), eGFRratio = eGFRcys(2012)/eGFRcr(2021), HR = hazard ratio, pSMI = predicted skeletal muscle index, SD = standard deviation, SI = sarcopenia index (Cr × eGFRcys), TBMM = total body muscle mass.

### 3.4. Supportive external analysis in NHANES

To explore cross-population consistency, we conducted a supportive external analysis using the NHANES 1999 to 2004 cohort (Tables S7, S8 and S9, Supplemental Digital Content 8). Overall, the protective directions of the 6 biomarkers were broadly consistent with those observed in CHARLS. However, constrained by smaller event counts, complex survey weighting, and cohort differences, the exact internal ordering and pairwise separation of the biomarkers were less precisely resolved in this secondary analysis, particularly in the presence of sex-related heterogeneity. Separately, IPW sensitivity analyses for complete-case inclusion are summarized in Table S10, Supplemental Digital Content 11, and did not materially alter the primary comparative interpretation.

## 4. Discussion

This study was designed to clarify comparative ordering across 6 related creatinine–cystatin C biomarkers within a correlated biomarker family. Taken together, the findings suggest that these biomarkers are better viewed as a correlated family conveying largely overlapping prognostic information, within which SI most consistently retained the clearest comparative signal. In single-biomarker analyses, SI, pSMI, and TBMM showed the most consistent inverse associations with all-cause mortality, whereas CCR, eGFRratio, and eGFRdiff were comparatively weaker, particularly in women. The spline analyses further showed steeper increases in mortality risk toward the lower ranges of the biomarkers, with comparatively flatter gradients at higher values (Fig. 2). In the prespecified mutually adjusted bootstrap framework, SI ranked highest in both sexes, whereas discrimination differences across single-biomarker models were small, and the joint 6-biomarker model provided only modest incremental improvement beyond standard covariates. These patterns indicate that the principal contribution of the study is to clarify comparative ordering within a highly correlated biomarker family, while gains in conventional predictive performance appear modest.

Viewed from that perspective, SI emerged as the clearest comparative signal within this biomarker family. This does not imply that SI can be equated with a direct measure of muscle mass; rather, it may more stably retain a muscle-related prognostic signal within a mixed filtration/non-GFR biomarker family. Ballew et al proposed creatinine filtration, operationalized as serum creatinine multiplied by cystatin C-based eGFR, as a physiologically motivated index related to frailty and mortality, while explicitly acknowledging the absence of direct muscle-mass validation and the potential influence of other determinants of creatinine filtration.^[13]^ More recently, Groothof et al showed that serum creatinine is strongly positively associated with muscle mass, whereas cystatin C is minimally associated with muscle mass and behaves differently after mutual adjustment; notably, when both markers were analyzed together, higher creatinine became associated with lower mortality, while the positive association between cystatin C and mortality strengthened.^[31]^ Within that context, combining creatinine with eGFRcys may help contextualize apparently low creatinine values in individuals with reduced muscle reserve, in whom creatinine alone may underestimate vulnerability. Where direct assessment of muscle-related impairment is not feasible, SI may serve as a pragmatic marker in this context. Accordingly, our findings support interpreting SI as the clearest comparative signal within this biomarker family under the present analytic framework, rather than as proof of biological primacy, a replacement for direct phenotyping, or evidence of major predictive superiority. Clinically, the modest incremental discrimination indicates that SI should not be used as a standalone mortality prediction tool or as a substitute for established clinical assessment. Rather, its potential value lies in providing complementary laboratory-derived information that may help characterize physiological vulnerability when interpreted together with age, comorbidity, kidney function, and, where available, direct measures of muscle mass, strength, and physical performance. Whether this complementary information improves clinical decision-making requires prospective external validation and evaluation against clinically relevant decision thresholds.

Furthermore, exploratory results in the NHANES subsample (Table S7, Supplemental Digital Content 8) provided preliminary evidence that the prognostic signal of SI persisted after additional adjustment for DXA-derived low muscle mass in the DXA subset. While these secondary findings were limited by sample size and heterogeneity, they suggest that these biomarkers may capture physiological vulnerability beyond simple quantitative muscle loss, while still requiring dedicated validation against direct muscle phenotyping and measured kidney function.

Although CCR, eGFRratio, and eGFRdiff were comparatively weaker in the present analyses, this should not be taken to mean that these markers are uninformative. Prior studies have linked multiple creatinine–cystatin C serum indices to mortality in disease-specific settings,^[20]^ and recent general-population evidence indicates that eGFR discordance itself carries prognostic information.^[21]^ However, the present study asked a narrower and more demanding question: which marker most consistently retains the clearest signal when 6 closely related biomarkers are evaluated side by side under mutual adjustment. In this setting, ratio- and discordance-based indices may still carry prognostic information, but they did not retain as stable a comparative signal under mutual adjustment within this highly correlated biomarker family. This interpretation is also consistent with recent comparative work suggesting that although eGFRratio is associated with risk, its incremental value is limited when more informative cystatin C-based measures are already available.^[32]^ The sign inversion observed for CCR after mutual adjustment is therefore better understood as a consequence of severe collinearity and conditional reallocation of shared variance than as evidence of harm.^[33,34]^ Such reversals are expected when highly correlated variables are entered into the same model and should be interpreted as conditional statistical signals rather than isolated biological effects.

This relative attenuation of CCR-, eGFRratio-, and eGFRdiff-based signals was more evident among women, whereas SI remained top-ranked in both sexes. This sex-stratified pattern is unlikely to be entirely explained by chance, because external evidence suggests that creatinine- and cystatin C-based kidney measures may differ in their risk expression by sex, likely reflecting sex-related differences in body composition and the non-GFR determinants of these biomarkers.^[14]^ The NHANES analyses were retained as exploratory supportive analyses rather than as a formal validation layer. Although the broader direction of associations was generally compatible with the CHARLS findings, the smaller event counts, survey design, and cohort differences limited precise resolution of biomarker ordering. These findings therefore provide external contextualization rather than evidence of transportability of absolute estimates or definitive comparative ranking.

Several limitations should be acknowledged. First, complete-case analyses may introduce selection bias, although IPW did not materially alter the primary ordering pattern. Second, we could not disentangle the relative contributions of filtration-related and non-GFR determinants to each biomarker, nor validate them against direct muscle imaging or measured GFR. This limitation is particularly relevant because creatinine filtration-type constructs may be influenced not only by muscle mass but also by diet, tubular secretion, extrarenal elimination of creatinine, and non-GFR determinants of cystatin C.^[13,31]^ Third, biomarkers were measured at baseline only, so longitudinal trajectories were not assessed. This is important because recent CHARLS-based evidence suggests that dynamic changes in cystatin C add prognostic information beyond baseline values alone.^[35]^ Fourth, while we attempted a supportive external analysis in NHANES, this was not a formal replication because of the relatively small number of mortality events and important differences in cohort design. The heterogeneity observed in the NHANES female subsample suggests that the exact comparative ranking of these biomarkers may vary across populations and clinical settings. Finally, the incremental performance estimates were paired in-sample summaries rather than out-of-sample validation. These limitations reinforce that the present findings should be interpreted as evidence of comparative prognostic ordering within a prespecified analytic framework rather than as a definitive biological decomposition of these biomarkers or as evidence of marked predictive superiority.

## 5. Conclusion

In older Chinese adults from CHARLS, 6 related creatinine–cystatin C biomarkers conveyed largely overlapping prognostic information in relation to all-cause mortality. Within this correlated biomarker set, SI showed the most consistent comparative association pattern and the highest ranking stability in the joint model, whereas differences in conventional discrimination were small and incremental improvement beyond standard covariates was modest. These findings support SI as the clearest comparative signal within this biomarker family rather than as evidence of marked predictive superiority.

## Acknowledgments

We thank the CHARLS team for data access. ChatGPT (OpenAI) and Paperpal were used for language editing and grammatical corrections to improve the readability of the manuscript. These tools were not used for data collection, statistical analysis, creation of results, or interpretation of findings. The authors reviewed all AI-assisted suggestions and take full responsibility for the final manuscript.

## Author contributions

**Conceptualization:** Ya Liu, Shuzhong Duan.

**Data curation:** Ya Liu, Yingsong Qi, Jin Zhou.

**Formal analysis:** Ya Liu, Yingsong Qi, Huanhuan Wang.

**Funding acquisition:** Ya Liu, Shuzhong Duan.

**Investigation:** Jin Zhou, Xiaojing Zhao, Min Zhang, Ce Liu, Jie Shen.

**Methodology:** Ya Liu, Yingsong Qi, Huanhuan Wang.

**Project administration:** Shuzhong Duan.

**Supervision:** Shuzhong Duan.

**Visualization:** Yingsong Qi.

**Writing – original draft:** Ya Liu, Yingsong Qi.

**Writing – review & editing:** Jin Zhou, Xiaojing Zhao, Min Zhang, Ce Liu, Jie Shen, Shuzhong Duan.

























## References

[1] Cruz-Jentoft AJ Bahat G Bauer J. Sarcopenia: revised European consensus on definition and diagnosis. Age Ageing. 2019;48:16–31. 10.1093/ageing/afy169 PMC6322506 30312372

[2] Chen LK Woo J Assantachai P. Asian Working Group for Sarcopenia: 2019 consensus update on sarcopenia diagnosis and treatment. J Am Med Dir Assoc. 2020;21:300–7.e2. 10.1016/j.jamda.2019.12.012 32033882

[3] Li R Xia J Zhang X. Associations of muscle mass and strength with all-cause mortality among US older adults. Med Sci Sports Exerc. 2018;50:458–67. 10.1249/MSS.0000000000001448 PMC5820209 28991040

[4] Yuan S Larsson SC. Epidemiology of sarcopenia: prevalence, risk factors, and consequences. Metabolism. 2023;144:155533. 10.1016/j.metabol.2023.155533 36907247

[5] Bernabei R Landi F Calvani R. Multicomponent intervention to prevent mobility disability in frail older adults: randomised controlled trial (SPRINTT project). BMJ. 2022;377:e068788. 10.1136/bmj-2021-068788 PMC9092831 35545258

[6] Calvani R Marini F Cesari M. Biomarkers for physical frailty and sarcopenia: state of the science and future developments. J Cachexia Sarcopenia Muscle. 2015;6:278–86. 10.1002/jcsm.12051 PMC4670735 26675566

[7] Lien YH. Looking for sarcopenia biomarkers. Am J Med. 2017;130:502–3. 10.1016/j.amjmed.2017.01.018 28189468

[8] Shlipak MG Matsushita K Ärnlöv J. Cystatin C versus creatinine in determining risk based on kidney function. N Engl J Med. 2013;369:932–43. 10.1056/NEJMoa1214234 PMC3993094 24004120

[9] Knight EL Verhave JC Spiegelman D. Factors influencing serum cystatin C levels other than renal function and the impact on renal function measurement. Kidney Int. 2004;65:1416–21. 10.1111/j.1523-1755.2004.00517.x 15086483

[10] Stevens LA Schmid CH Greene T. Factors other than glomerular filtration rate affect serum cystatin C levels. Kidney Int. 2009;75:652–60. 10.1038/ki.2008.638 PMC4557800 19119287

[11] Baxmann AC Ahmed MS Marques NC. Influence of muscle mass and physical activity on serum and urinary creatinine and serum cystatin C. Clin J Am Soc Nephrol. 2008;3:348–54. 10.2215/CJN.02870707 PMC2390952 18235143

[12] Kidney Disease: Improving Global Outcomes (KDIGO) CKD Work Group. KDIGO 2024 clinical practice guideline for the evaluation and management of chronic kidney disease. Kidney Int. 2024;105:S117–314. 10.1016/j.kint.2023.10.018 38490803

[13] Ballew SH Zhou L Surapaneni A. A novel creatinine muscle index based on creatinine filtration: associations with frailty and mortality. J Am Soc Nephrol. 2023;34:495–504. 10.1681/ASN.0000000000000037 PMC10103307 36735317

[14] De La Mata NL Hedley JA Webster AC. Sex differences in cardiac risk and kidney function: serum creatinine versus cystatin C. BMC Med. 2025;24:62. 10.1186/s12916-025-04588-9 PMC12860083 41462300

[15] Kusunoki H Tabara Y Tsuji S. Estimation of muscle mass using creatinine/cystatin C ratio in Japanese community-dwelling older people. J Am Med Dir Assoc. 2022;23:902.e21–31. 10.1016/j.jamda.2021.07.029 34437868

[16] Kim SW Jung HW Kim CH Kim KI Chin HJ Lee H. A new equation to estimate muscle mass from creatinine and cystatin C. PLoS One. 2016;11:e0148495. 10.1371/journal.pone.0148495 PMC4744004 26849842

[17] Liu Y Qi Y Wang H. Predicted skeletal muscle index as a scalable marker for sarcopenia risk and mortality in older Chinese adults. Front Med (Lausanne). 2025;12:1640410. 10.3389/fmed.2025.1640410 PMC12405267 40909453

[18] Yamada Y Nakashima H Nagae M. Association of serum creatinine-cystatin C ratio with hospital-associated complications and post-discharge mortality. Geriatr Gerontol Int. 2025;25:1564–70. 10.1111/ggi.70206 PMC12584936 41031546

[19] Wang S Yang T Bao Y. Association of serum creatinine-cystatin C ratio with all-cause, cardiovascular and cancer mortality in US adults: a nationwide cohort study. BMC Geriatr. 2024;24:963. 10.1186/s12877-024-05546-5 PMC11577820 39567886

[20] Lin YL Chang IC Liou HH. Serum indices based on creatinine and cystatin C predict mortality in patients with non-dialysis chronic kidney disease. Sci Rep. 2021;11:16863. 10.1038/s41598-021-96447-9 PMC8377030 34413438

[21] Liu Q Celis-Morales C Lees JS. Discordance between cystatin C-based and creatinine-based estimated glomerular filtration rate and mortality in the general population. Clin Chem. 2025;71:858–69. 10.1093/clinchem/hvaf063 40464748

[22] Chen DC Shlipak MG Scherzer R. Association of intraindividual difference in estimated glomerular filtration rate by creatinine vs cystatin C and end-stage kidney disease and mortality. JAMA Netw Open. 2022;5:e2148940. 10.1001/jamanetworkopen.2021.48940 PMC8855239 35175342

[23] Carrero JJ Fu EL Sang Y. Discordances between creatinine- and cystatin C-based estimated GFR and adverse clinical outcomes in routine clinical practice. Am J Kidney Dis. 2023;82:534–42. 10.1053/j.ajkd.2023.04.002 37354936

[24] Zhao Y Hu Y Smith JP Strauss J Yang G. Cohort profile: the China Health and Retirement Longitudinal Study (CHARLS). Int J Epidemiol. 2014;43:61–8. 10.1093/ije/dys203 PMC3937970 23243115

[25] Inker LA Schmid CH Tighiouart H. Estimating glomerular filtration rate from serum creatinine and cystatin C. N Engl J Med. 2012;367:20–9. 10.1056/NEJMoa1114248 PMC4398023 22762315

[26] Inker LA Eneanya ND Coresh J. New creatinine- and cystatin C-based equations to estimate GFR without race. N Engl J Med. 2021;385:1737–49. 10.1056/NEJMoa2102953 PMC8822996 34554658

[27] Wen X Wang M Jiang CM Zhang YM. Anthropometric equation for estimation of appendicular skeletal muscle mass in Chinese adults. Asia Pac J Clin Nutr. 2011;20:551–6. 22094840

[28] Zhong BX Zhong HL Zhou GQ Xu WQ Lu Y Zhao QF. Physical performance and risk of hip fracture in community-dwelling elderly people in China: a 4-year longitudinal cohort study. Maturitas. 2021;146:26–33. 10.1016/j.maturitas.2021.01.003 33722361

[29] Grambsch PM Therneau TM. Proportional hazards tests and diagnostics based on weighted residuals. Biometrika. 1994;81:515–26.

[30] Shi S Chen W Jiang Y Chen K Liao Y Huang K. A more accurate method to estimate muscle mass: a new estimation equation. J Cachexia Sarcopenia Muscle. 2023;14:1753–61. 10.1002/jcsm.13254 PMC10401528 37203296

[31] Groothof D Shehab NBN Erler NS. Creatinine, cystatin C, muscle mass, and mortality: findings from a primary and replication population-based cohort. J Cachexia Sarcopenia Muscle. 2024;15:1528–38. 10.1002/jcsm.13511 PMC11294032 38898741

[32] Tydén M Batra G Fellström B. Different eGFR markers and prediction of cardiovascular risk. J Intern Med. 2026;299:143–57. 10.1111/joim.70050 PMC12678217 41273211

[33] Kim JH. Multicollinearity and misleading statistical results. Korean J Anesthesiol. 2019;72:558–69. 10.4097/kja.19087 PMC6900425 31304696

[34] MacKinnon DP Krull JL Lockwood CM. Equivalence of the mediation, confounding and suppression effect. Prev Sci. 2000;1:173–81. 10.1023/a:1026595011371 PMC2819361 11523746

[35] Zhang Y Zhang L Xing J. Longitudinal changes in plasma Cystatin C and all-cause mortality risk among the middle-aged and elderly Chinese population. Clin Biochem. 2025;135:110858. 10.1016/j.clinbiochem.2024.110858 39581251

