# Supplementary material for: Comparative evaluation of the sarcopenia index and 5 related creatinine–cystatin C biomarkers in relation to all-cause mortality in older adults: A cohort study
Source: Medicine (Baltimore). 2026 Oct 2;105(40):e50842. doi: 10.1097/MD.0000000000050842 (PMC13632987; doi:10.1097/MD.0000000000050842)
Supplement: Supplementary file 1 [file medi-105-e50842-s001.pdf]

Supplementary Methods

S1. Locked sample definition

We used a locked complete-case strategy within each sex. For comparative association analyses and joint-model analyses, we restricted analyses to a locked intersection sample with complete data for the time-to-event outcome (time and event), the Model 2 covariates, and all six primary biomarkers. For incremental discrimination comparisons, locked samples were defined per comparison (time-to-event outcome, baseline covariates, and the biomarker set required for that comparison) so that baseline and augmented models were fit and evaluated on the same participants.

S2. Best probability bootstrap procedure (joint model conditional ranking)

Within each sex, we fitted a Model 2-adjusted joint Cox model simultaneously including the six biomarkers (entered as sex-specific z-scores). Models were fitted within the locked intersection complete-case sample defined in S1. To summarize conditional dominance under severe multicollinearity, we performed nonparametric bootstrap resampling of participants with replacement (B = 1000). In each bootstrap replicate, the same joint model was refitted and the six biomarker coefficients (for z-scored biomarkers) were extracted. For each replicate, the "most protective" biomarker was defined as the biomarker with the smallest (most negative) coefficient among those with finite estimates in that replicate. For each biomarker, Best probability was computed as the proportion of bootstrap replicates in which that biomarker was most protective; replicates without a finite estimate for a given biomarker were excluded from that biomarker's denominator (NonNA), defined as the number of bootstrap replicates with finite estimates for that biomarker. In the present analyses, all six biomarkers had NonNA = 1000; therefore, Best probability denominators did not differ across biomarkers. Pairwise probabilities P($\beta_i < \beta_j$) were computed empirically across bootstrap replicates with finite estimates for both biomarkers. Pairwise coefficient contrasts in the original (non-bootstrapped) joint model were evaluated using Wald tests for $\beta_i - \beta_j$, with Benjamini-Hochberg FDR adjustment within sex.

S3. Optimism-corrected internal performance assessment via bootstrap optimism correction (Table 4)

This section reports optimism-corrected internal performance metrics for the fully adjusted (Model 2) single-biomarker Cox models and should be distinguished from the paired in-sample apparent discrimination differences summarized in S4. Internal performance assessment used bootstrap optimism correction (B = 200) via nonparametric resampling of participants with replacement. In each bootstrap resample, the model was refitted and performance was computed in the bootstrap sample (apparent) and in the original locked sample (test) using the bootstrap-fitted model. Optimism was defined as performance (bootstrap-fit in bootstrap sample) − performance (bootstrap-fit in original sample). Optimism-corrected performance was computed as performance (original-fit model in original sample)

– mean optimism. For each biomarker, Table 4 additionally reports optimism-corrected discrimination (Harrell's C-index), 5-year calibration metrics (Brier score, calibration-in-the-large, calibration slope), and optimism-corrected ΔC-index versus the covariates-only baseline.

S4. Apparent paired incremental discrimination via event-stratified bootstrap (Supplementary Table S5)

This section reports paired in-sample (apparent) discrimination differences beyond the covariates-only baseline and should not be interpreted as optimism-corrected performance estimates. Baseline and augmented models were refitted on the same locked sample and compared using paired event-stratified bootstrap resampling (B = 1000), in which deaths and non-deaths were resampled separately with replacement and then combined. In each replicate, both models were refitted and the paired differences in ΔC-index and, where reported, ΔAUC_5y were computed within each bootstrap resample; percentile 95% confidence intervals were obtained from the empirical distributions of these paired differences. Time-dependent AUC at 5 years (AUC_5y) was estimated using an IPCW time-dependent AUC (Uno-type). AUC_5y was evaluated just before 5 years, when necessary, to avoid boundary instability at the administrative censoring time.

S5. Supportive external analysis in NHANES (1999–2004)

For the supportive external analysis, we used the combined NHANES 1999–2004 cycles among adults aged 60 years or older in the cystatin C subsample. We constructed the analytic dataset by merging demographics, examination, laboratory, and linked mortality files by participant identifier (SEQN). Follow-up time was derived from person-months since examination and converted to years; follow-up was administratively censored at 5 years by truncating time at 5 years and defining events as deaths occurring within the 5-year horizon. Survey-weighted analyses used the cystatin C subsample weights; for the combined 1999–2004 cycles, the 2-year subsample weights were scaled by 1/3 to form a 6-year combined weight. Sex-stratified survey-weighted Cox models were fitted using the complex survey design variables (strata and PSUs). Sex-specific z-scores were computed using survey-weighted means and standard deviations. To ensure fair comparative analyses across biomarkers, analyses were restricted to a locked intersection complete-case sample with complete data for the time-to-event outcome, survey design variables and weights, the covariate set defined as Model 2, and all six biomarkers. Biomarkers were standardized within sex as z-scores, and hazard ratios were reported per 1-SD increase. Covariate adjustment otherwise paralleled CHARLS where available; however, because public NHANES does not provide geographic-context variables comparable to the CHARLS area measure, NHANES models additionally adjusted for poverty-to-income ratio (INDFMPIR) as an available indicator of socioeconomic context. As a sensitivity analysis, an NHANES-derived estimated appendicular skeletal muscle mass (ASM) proxy was evaluated using the published equation from Shi et al. [30]:

$$ASM = 0.595 \times 0.998^{Age} \times 0.825^{Female} \times 1.079^{Black} \times 1.005^{Height} \times Weight^{0.677} \times Cr^{0.143} \times CysC^{-0.104}$$

where Age is in years; Height, in cm; Weight, in kg; Cr, in mg/dL; and CysC, in mg/L. Female was coded as 1 for women and 0 for men, and *Black* as 1 for non-Hispanic Black participants and 0 otherwise. Given the limited number of deaths in the cystatin C subsample and the complex survey design, NHANES results were interpreted primarily as supportive external evidence rather than as formal external validation or transportability assessment.

S6. IPW sensitivity analysis for inclusion

As a sensitivity analysis for potential selection bias related to complete-case inclusion, we performed inverse probability weighting (IPW) for inclusion in the final analytic sample (Supplementary Table S10). Inclusion models were fitted separately by sex in the combined included/excluded CHARLS 2015 sample using logistic regression with baseline variables available in both groups, including age, residence, marital status, smoking, drinking, lung disease, hypertension, diabetes, cancer, heart disease, stroke, psychiatric disease, liver disease, kidney disease, and joint disease. Stabilized inclusion weights were trimmed at the sex-specific 1st and 99th percentiles and then applied to the final sex-specific complete-case Cox models. We repeated the Model 2 single-biomarker analyses for all six primary biomarkers and the six-biomarker joint-model bootstrap ranking analysis under IPW (B = 1000). These analyses assessed whether differential inclusion materially altered the primary comparative biomarker ordering.

S7. Software and reproducibility

Analyses were conducted in R (version 4.4.3) using the survival, glmnet, timeROC, and survAUC packages. Package versions were managed with renv. Prespecified seeds were used for resampling-based procedures; script-level seeds and replicate counts are reported alongside outputs where applicable.
