# Supplementary material for: Comparative evaluation of the sarcopenia index and 5 related creatinine–cystatin C biomarkers in relation to all-cause mortality in older adults: A cohort study
Source: Medicine (Baltimore). 2026 Oct 2;105(40):e50842. doi: 10.1097/MD.0000000000050842 (PMC13632987; doi:10.1097/MD.0000000000050842)
Supplement: Supplementary file 2 [file medi-105-e50842-s002.pdf]

Supplementary Table S1. Baseline characteristics by inclusion status and sex

Characteristics	Included Men (N = 2987)	Excluded Men (N = 1871)	p-value (Men)	Included Women (N = 2995)	Excluded Women (N = 1992)	p-value (Women)
Age, years	68.130 (6.432)	68.897 (7.351)	<0.001	67.802 (6.508)	69.721 (8.207)	<0.001
Residence area			<0.001			<0.001
Rural Village	1,929 (64.6%)	1,031 (55.1%)		1,882 (62.8%)	1,082 (54.3%)	
Urban Community	1,058 (35.4%)	840 (44.9%)		1,113 (37.2%)	910 (45.7%)	
Marital status			0.004			<0.001
Married	2,489 (83.3%)	1,497 (80.0%)		2,144 (71.6%)	1,263 (63.4%)	
Single	498 (16.7%)	374 (20.0%)		851 (28.4%)	729 (36.6%)	
Smoking	1,501 (50.3%)	822 (44.4%)	<0.001	179 (6.0%)	136 (6.9%)	0.216
Missing	0	18		0	19	
Alcohol drinking	2,139 (71.6%)	1,281 (69.6%)	0.141	691 (23.1%)	454 (23.2%)	0.937
Missing	0	30		0	36	
Weight, kg	60.829 (11.106)	60.510 (12.333)	0.477	54.632 (10.284)	53.545 (12.317)	0.012
Missing	0	922		0	979	
Height, cm	162.668 (6.697)	162.216 (7.677)	0.106	150.914 (6.066)	150.097 (10.039)	0.015
Missing	0	935		0	984	
WHtR	0.525 (0.061)	0.525 (0.085)	0.958	0.577 (0.070)	0.578 (0.190)	0.858
Missing	0	939		0	989	

Characteristics	Included Men (N = 2987)	Excluded Men (N = 1871)	p-value (Men)	Included Women (N = 2995)	Excluded Women (N = 1992)	p-value (Women)
SMI, kg/m²	7.473 (0.698)	7.479 (0.981)	0.862	5.777 (0.825)	5.714 (1.524)	0.207
Missing	0	941		0	993	
Grip strength, kg	34.417 (7.893)	32.980 (8.756)	<0.001	23.062 (6.225)	21.235 (6.716)	<0.001
Missing	28	910		61	978	
Physical performance			<0.001			<0.001
Abnormal	375 (13.5%)	176 (20.9%)		648 (23.6%)	308 (35.7%)	
Normal	2,411 (86.5%)	666 (79.1%)		2,102 (76.4%)	554 (64.3%)	
Missing	201	1,029		245	1,130	
Hemoglobin, g/dL	14.247 (1.763)	14.643 (2.065)	0.002	12.855 (1.593)	13.002 (1.833)	0.174
Missing	0	1,588		0	1,681	
Triglycerides, mg/dL	98.230 [74.336, 143.363]	105.310 [75.221, 153.982]	0.081	123.894 [90.265, 178.761]	136.283 [96.460, 186.726]	0.024
Missing	0	1,558		0	1,657	
hs-CRP, mg/L	1.400 [0.800, 2.700]	1.600 [0.900, 3.200]	0.014	1.500 [0.800, 2.800]	1.600 [0.900, 3.400]	0.192
Missing	0	1,559		0	1,657	
Serum creatinine, mg/dL	0.890 [0.787, 1.020]	0.879 [0.775, 0.993]	0.045	0.696 [0.619, 0.790]	0.702 [0.609, 0.828]	0.327
Missing	0	1,558		0	1,657	

Characteristics	Included Men (N = 2987)	Excluded Men (N = 1871)	p-value (Men)	Included Women (N = 2995)	Excluded Women (N = 1992)	p-value (Women)
Cystatin C, mg/L	0.920 [0.800, 1.030]	0.910 [0.800, 1.020]	0.834	0.870 [0.760, 0.980]	0.910 [0.790, 1.010]	0.002
Missing	0	1,560		0	1,659	
LDL cholesterol, mg/dL	98.504 (27.733)	99.222 (30.015)	0.686	108.758 (29.441)	107.098 (32.503)	0.372
Missing	0	1,559		0	1,657	
CCR	0.980 [0.874, 1.097]	0.964 [0.843, 1.082]	0.078	0.808 [0.718, 0.912]	0.783 [0.689, 0.903]	0.025
Missing	0	1,560		0	1,659	
SI	74.926 [65.226, 84.756]	73.867 [62.275, 84.063]	0.151	58.053 [50.607, 65.401]	56.288 [47.766, 64.648]	0.008
Missing	0	1,560		0	1,659	
pSMI	7.513 (0.760)	7.662 (0.921)	0.022	6.295 (0.663)	6.431 (0.845)	0.017
Missing	0	1,658		0	1,757	
TBMM	42.377 [38.158, 46.797]	42.902 [38.875, 46.470]	0.211	32.136 [29.271, 35.086]	32.644 [29.856, 35.571]	0.207
Missing	0	1,618		0	1,719	
eGFRcys (CKD-EPI 2012), mL/min/1.73m²	84.395 (19.992)	84.428 (19.719)	0.978	83.192 (18.262)	79.640 (20.178)	0.002
Missing	0	1,560		0	1,659	
eGFRcr (CKD-EPI 2021),	87.135 (15.879)	88.376 (14.823)	0.162	87.705 (15.967)	86.222 (16.425)	0.117

Characteristics	Included Men (N = 2987)	Excluded Men (N = 1871)	p-value (Men)	Included Women (N = 2995)	Excluded Women (N = 1992)	p-value (Women)
mL/min/1.73m²						
Missing	0	1,558		0	1,657	
eGFRratio	0.966 [0.841, 1.077]	0.960 [0.829, 1.069]	0.325	0.956 [0.834, 1.045]	0.939 [0.804, 1.034]	0.036
Missing	0	1,560		0	1,659	
eGFRdiff	-2.740 (16.277)	-3.918 (18.377)	0.277	-4.513 (15.583)	-6.625 (13.959)	0.010
Missing	0	1,560		0	1,659	
Hypertension	1,183 (39.6%)	675 (38.0%)	0.295	1,337 (44.6%)	861 (44.9%)	0.874
Missing	0	96		0	75	
Diabetes	303 (10.1%)	192 (10.9%)	0.464	424 (14.2%)	261 (13.7%)	0.679
Missing	0	103		0	86	
Lung disease	597 (20.0%)	385 (21.7%)	0.171	412 (13.8%)	310 (16.2%)	0.020
Missing	0	96		0	80	
Heart disease	575 (19.3%)	350 (19.8%)	0.700	752 (25.1%)	520 (27.2%)	0.113
Missing	0	99		0	79	
Stroke	167 (5.6%)	148 (8.4%)	<0.001	127 (4.2%)	126 (6.6%)	<0.001
Missing	0	101		0	80	
Liver disease	209 (7.0%)	122 (6.9%)	0.946	185 (6.2%)	108 (5.7%)	0.501

Characteristics	Included Men (N = 2987)	Excluded Men (N = 1871)	p-value (Men)	Included Women (N = 2995)	Excluded Women (N = 1992)	p-value (Women)
Missing	0	103		0	86	
Kidney disease	362 (12.1%)	229 (13.0%)	0.418	249 (8.3%)	197 (10.3%)	0.019
Missing	0	105		0	84	
Cancer	41 (1.4%)	28 (1.6%)	0.645	62 (2.1%)	44 (2.3%)	0.650
Missing	0	102		0	84	
Digestive disease	815 (27.3%)	507 (28.5%)	0.364	1,063 (35.5%)	675 (35.3%)	0.927
Missing	0	95		0	81	
Joint disease	1,214 (40.6%)	721 (40.6%)	0.987	1,619 (54.1%)	1,024 (53.5%)	0.725
Missing	0	94		0	78	
Dyslipidemia	569 (19.0%)	315 (18.0%)	0.392	707 (23.6%)	366 (19.3%)	<0.001
Missing	0	121		0	94	

Data are presented as mean ± SD for normally distributed variables, median (IQR) for non-normally distributed variables, and n (%) for categorical variables. p-values were calculated using t-tests for normally distributed continuous variables, Wilcoxon rank-sum tests for non-normally distributed continuous variables, and chi-square tests for categorical variables. Missing indicates number of missing values in each group. WHtR, waist-to-height ratio; SMI, skeletal muscle index; CCR, creatinine-to-cystatin C ratio; pSMI, predicted skeletal muscle index; SI, sarcopenia index (Cr × eGFRcys); eGFRratio, eGFRcys(2012)/eGFRcr(2021); eGFRdiff, eGFRcys(2012) − eGFRcr(2021); TBMM, total body muscle mass.
