# Supplementary material for: Comparative evaluation of the sarcopenia index and 5 related creatinine–cystatin C biomarkers in relation to all-cause mortality in older adults: A cohort study
Source: Medicine (Baltimore). 2026 Oct 2;105(40):e50842. doi: 10.1097/MD.0000000000050842 (PMC13632987; doi:10.1097/MD.0000000000050842)
Supplement: Supplementary file 3 [file medi-105-e50842-s003.pdf]

Supplementary Table S2. Biomarker definitions, component equations, input units, and source references

Biomarker	Definition / equation	Input units	Source reference(s)
CCR	CCR = Cr / CysC	Cr in mg/dL; CysC in mg/L	[15]
eGFRcys(2012)	eGFRcys(2012) = $133 \times \min(\text{CysC}/0.8, 1)^{-0.499} \times \max(\text{CysC}/0.8, 1)^{-1.328} \times 0.996^{\text{Age}} \times 0.932$ (if female)	CysC in mg/L; Age in years	[25]
eGFRcr(2021)	eGFRcr(2021) = $142 \times \min(\text{Cr}/\kappa, 1)^{\alpha} \times \max(\text{Cr}/\kappa, 1)^{-1.200} \times 0.9938^{\text{Age}} \times 1.012$ (if female), where $\kappa = 0.7$ and $\alpha = -0.241$ for women, and $\kappa = 0.9$ and $\alpha = -0.302$ for men	Cr in mg/dL; Age in years	[26]
SI	SI = Cr × eGFRcys(2012)	Cr in mg/dL; CysC in mg/L; Age in years	[13,25]
eGFRratio	eGFRratio = eGFRcys(2012) / eGFRcr(2021)	Cr in mg/dL; CysC in mg/L; Age in years	[25,26]
eGFRdiff	eGFRdiff = eGFRcys(2012) − eGFRcr(2021)	Cr in mg/dL; CysC in mg/L; Age in years	[25,26]
pSMI	Men: pSMI = 4.17 − 0.012 × Age + 1.24 × (Cr/CysC) − 0.0513 × Hb + 0.0598 × Weight<br>Women: pSMI = 3.55 − 0.00765 × Age + 0.852 × (Cr/CysC) − 0.0627 × Hb + 0.0614 × Weight	Age in years; Cr in mg/dL; CysC in mg/L; Hb in g/dL; Weight in kg	[17]
TBMM	Men: TBMM = (Weight × Cr) / (0.00675 × Weight × CysC + Cr)<br>Women: TBMM = (Weight × Cr) / (0.01006 × Weight × CysC + Cr)	Weight in kg; Cr in mg/dL; CysC in mg/L	[16]

Abbreviations: CCR, creatinine-to-cystatin C ratio; SI, sarcopenia index (Cr × eGFRcys); pSMI, predicted skeletal muscle index; TBMM, total body muscle mass; Cr, creatinine; CysC, cystatin C; Hb, hemoglobin.
