# Supplementary material for: Comparative evaluation of the sarcopenia index and 5 related creatinine–cystatin C biomarkers in relation to all-cause mortality in older adults: A cohort study
Source: Medicine (Baltimore). 2026 Oct 2;105(40):e50842. doi: 10.1097/MD.0000000000050842 (PMC13632987; doi:10.1097/MD.0000000000050842)
Supplement: Supplementary file 4 [file medi-105-e50842-s004.pdf]

Supplementary Table S3. Sex-specific quartile associations of six related creatinine-cystatin C biomarkers with all-cause mortality in CHARLS 2015–2020

Biomarker	Category	Men				Women			
		N = 2987, Death = 428				N = 2995, Death = 228			
		Unadjusted	Model 1	Model 2	Model 3†	Unadjusted	Model 1	Model 2	Model 3†
CCR	Q1 (lowest, reference)	1.00 (reference)	1.00 (reference)	1.00 (reference)	1.00 (reference)	1.00 (reference)	1.00 (reference)	1.00 (reference)	1.00 (reference)
	Q2	0.64 (0.50–0.81)***	0.71 (0.55–0.90)**	0.74 (0.60–1.00)*	0.76 (0.59–1.00)*	0.63 (0.45–0.87)**	0.75 (0.54–1.04)	0.81 (0.57–1.20)	0.80 (0.56–1.14)
	Q3	0.44 (0.33–0.57)***	0.54 (0.41–0.71)***	0.60 (0.46–0.77)***	0.62 (0.48–0.83)***	0.40 (0.28–0.59)***	0.54 (0.37–0.79)**	0.57 (0.37–0.84)**	0.56 (0.37–0.81)**
	Q4	0.41 (0.31–0.54)***	0.55 (0.41–0.72)***	0.62 (0.47–0.86)***	0.64 (0.47–0.85)**	0.37 (0.25–0.55)***	0.57 (0.38–0.85)**	0.61 (0.41–0.95)*	0.62 (0.40–0.90)*
pSMI	Q1 (lowest, reference)	1.00 (reference)	1.00 (reference)	1.00 (reference)	1.00 (reference)	1.00 (reference)	1.00 (reference)	1.00 (reference)	1.00 (reference)
	Q2	0.53 (0.41–0.68)***	0.64 (0.50–0.83)***	0.72 (0.55–0.93)**	0.85 (0.62–1.06)	0.45 (0.32–0.64)***	0.67 (0.46–0.97)*	0.65 (0.42–1.00)*	0.83 (0.50–1.43)
	Q3	0.47 (0.36–0.61)***	0.62 (0.46–0.83)**	0.67 (0.51–0.94)**	0.83 (0.57–1.18)	0.46 (0.33–0.65)***	0.81 (0.55–1.20)	0.76 (0.49–1.22)	0.96 (0.57–1.76)
	Q4	0.35 (0.27–0.47)***	0.48 (0.33–0.69)***	0.51 (0.39–0.81)***	0.59 (0.37–0.92)**	0.27 (0.18–0.41)***	0.50 (0.31–0.82)**	0.47 (0.27–0.78)**	0.58 (0.31–1.14)
SI	Q1 (lowest, reference)	1.00 (reference)	1.00 (reference)	1.00 (reference)	1.00 (reference)	1.00 (reference)	1.00 (reference)	1.00 (reference)	1.00 (reference)
	Q2	0.52 (0.41–0.66)***	0.67 (0.52–0.85)**	0.73 (0.60–0.94)**	0.73 (0.55–0.93)*	0.42 (0.31–0.59)***	0.61 (0.43–0.85)**	0.62 (0.44–0.90)**	0.63 (0.44–0.88)**
	Q3	0.38 (0.29–	0.54 (0.41–	0.61 (0.45–	0.64 (0.48–	0.24 (0.16–	0.39 (0.26–	0.41 (0.27–	0.42 (0.27–

Biomarker	Category	Men				Women			
		N = 2987, Death = 428				N = 2995, Death = 228			
		Unadjusted	Model 1	Model 2	Model 3†	Unadjusted	Model 1	Model 2	Model 3†
		0.50)***	0.71)***	0.84)***	0.87)**	0.36)***	0.60)***	0.62)***	0.64)***
	Q4	0.30 (0.23–0.41)***	0.49 (0.36–0.66)***	0.57 (0.39–0.82)***	0.59 (0.42–0.79)***	0.23 (0.16–0.35)***	0.43 (0.28–0.67)***	0.46 (0.29–0.73)***	0.48 (0.30–0.73)**
eGFRratio	Q1 (lowest, reference)	1.00 (reference)	1.00 (reference)	1.00 (reference)	1.00 (reference)	1.00 (reference)	1.00 (reference)	1.00 (reference)	1.00 (reference)
	Q2	0.55 (0.43–0.70)***	0.63 (0.49–0.81)***	0.67 (0.54–0.94)**	0.68 (0.54–0.86)**	0.61 (0.44–0.84)**	0.79 (0.56–1.09)	0.81 (0.59–1.17)	0.82 (0.57–1.17)
	Q3	0.43 (0.33–0.56)***	0.57 (0.44–0.75)***	0.62 (0.46–0.81)***	0.63 (0.47–0.79)**	0.38 (0.26–0.55)***	0.56 (0.38–0.83)**	0.59 (0.40–0.94)*	0.60 (0.38–0.87)*
	Q4	0.39 (0.30–0.51)***	0.53 (0.40–0.70)***	0.60 (0.44–0.85)***	0.63 (0.44–0.78)**	0.38 (0.26–0.55)***	0.55 (0.37–0.81)**	0.58 (0.39–0.87)**	0.58 (0.38–0.86)**
eGFRdiff	Q1 (lowest, reference)	1.00 (reference)	1.00 (reference)	1.00 (reference)	1.00 (reference)	1.00 (reference)	1.00 (reference)	1.00 (reference)	1.00 (reference)
	Q2	0.72 (0.57–0.91)**	0.74 (0.58–0.94)*	0.78 (0.61–0.97)*	0.78 (0.62–1.00)*	0.86 (0.62–1.18)	1.00 (0.72–1.38)	1.06 (0.75–1.51)	1.04 (0.72–1.41)
	Q3	0.53 (0.41–0.69)***	0.65 (0.50–0.85)**	0.69 (0.53–0.96)**	0.70 (0.52–0.95)**	0.54 (0.37–0.78)**	0.73 (0.50–1.06)	0.78 (0.53–1.17)	0.78 (0.50–1.13)
	Q4	0.39 (0.29–0.52)***	0.52 (0.39–0.70)***	0.60 (0.46–0.81)***	0.62 (0.46–0.83)**	0.43 (0.29–0.64)***	0.61 (0.40–0.92)*	0.65 (0.41–1.03)	0.65 (0.39–0.97)*
TBMM	Q1 (lowest, reference)	1.00 (reference)	1.00 (reference)	1.00 (reference)	1.00 (reference)	1.00 (reference)	1.00 (reference)	1.00 (reference)	1.00 (reference)
	Q2	0.52 (0.40–0.67)***	0.58 (0.45–0.76)***	0.62 (0.49–0.88)**	0.80 (0.59–1.02)	0.43 (0.30–0.60)***	0.60 (0.42–0.87)**	0.60 (0.43–0.86)**	0.68 (0.44–1.01)

Biomarker	Category	Men				Women			
		N = 2987, Death = 428				N = 2995, Death = 228			
		Unadjusted	Model 1	Model 2	Model 3†	Unadjusted	Model 1	Model 2	Model 3†
	Q3	0.53 (0.41–0.68)***	0.63 (0.47–0.83)**	0.67 (0.50–0.90)*	0.87 (0.62–1.17)	0.40 (0.28–0.57)***	0.66 (0.45–0.97)*	0.65 (0.44–1.01)	0.76 (0.48–1.19)
	Q4	0.37 (0.28–0.50)***	0.48 (0.33–0.68)***	0.52 (0.39–0.90)***	0.63 (0.43–0.88)*	0.25 (0.16–0.37)***	0.46 (0.28–0.73)**	0.44 (0.26–0.76)***	0.50 (0.28–0.83)**

Model 2 is the primary adjustment set; Model 3† is a sensitivity analysis that additionally adjusted for the AWGS-aligned sarcopenia. Model 1 adjusted for age and waist-to-height ratio (WHtR). Model 2 additionally adjusted for residence area, smoking, drinking, lung disease, hypertension, diabetes, cancer, stroke, psychiatric disease, liver disease, kidney disease, joint disease, log triglycerides (logTG), and log C-reactive protein (logCRP). CHARLS, China Health and Retirement Longitudinal Study; CCR, creatinine-to-cystatin C ratio; pSMI, predicted skeletal muscle index; SI, sarcopenia index (Cr × eGFRcys); eGFRratio, eGFRcys(2012)/eGFRcr(2021); eGFRdiff, eGFRcys(2012) − eGFRcr(2021); TBMM, total body muscle mass; WHtR, waist-to-height ratio. *p<0.05, **p<0.01, ***p<0.001.
