# Supplementary material for: Comparative evaluation of the sarcopenia index and 5 related creatinine–cystatin C biomarkers in relation to all-cause mortality in older adults: A cohort study
Source: Medicine (Baltimore). 2026 Oct 2;105(40):e50842. doi: 10.1097/MD.0000000000050842 (PMC13632987; doi:10.1097/MD.0000000000050842)
Supplement: Supplementary file 5 [file medi-105-e50842-s005.pdf]

Supplementary Table S4. Pairwise comparisons of joint-model coefficients among six biomarkers, by sex in CHARLS 2015–2020

Comparison	Men			Women		
	N=2987; Death=428			N=2995; Death=228		
	Diff (βA − βB)	q(BH)	Pr(βA < βB)	Diff (βA − βB)	q(BH)	Pr(βA < βB)
SI vs pSMI	-0.704	0.069	97.5%	-1.313	0.005	99.5%
SI vs CCR	-1.403	<0.001	99.9%	-2.206	0.005	99.9%
SI vs eGFRratio	-0.977	0.028	99.3%	-1.664	0.016	99.7%
SI vs TBMM	-0.564	0.229	91.2%	-0.883	0.144	95.1%
SI vs eGFRdiff	-0.588	0.092	97.2%	-1.365	0.005	99.8%
pSMI vs CCR	-0.699	0.092	96.8%	-0.893	0.045	97.7%
pSMI vs eGFRratio	-0.273	0.636	72.7%	-0.351	0.547	77.6%
pSMI vs TBMM	0.140	0.850	42.7%	0.430	0.495	22.6%
pSMI vs eGFRdiff	0.117	0.738	32.3%	-0.052	0.796	64.5%
CCR vs eGFRratio	0.426	0.292	9.4%	0.542	0.418	17.5%
CCR vs TBMM	0.839	0.028	0.9%	1.322	0.016	1.1%
CCR vs eGFRdiff	0.815	0.002	0.0%	0.841	0.046	2.8%
eGFRratio vs TBMM	0.413	0.210	6.8%	0.781	0.019	1.7%
eGFRratio vs eGFRdiff	0.389	0.306	13.2%	0.299	0.552	25.4%
TBMM vs eGFRdiff	-0.023	0.948	48.7%	-0.482	0.380	85.6%

Diff (βA − βB) is the difference in standardized (z-score) coefficients between biomarker A and biomarker B from the mutually adjusted six-biomarker joint Cox model (sex-stratified; additionally adjusted for Model 2 covariates as defined in Table 2). q(BH) denotes Benjamini-Hochberg FDR-adjusted p-values for the 15 within-sex pairwise contrasts. Pr(βA < βB) is the bootstrap-estimated probability that βA is more protective (smaller coefficient) than βB, based on the joint-model bootstrap (B =

1000). SI, sarcopenia index (Cr × eGFRcys); pSMI, predicted skeletal muscle index; CCR, creatinine-to-cystatin C ratio; eGFRratio, eGFRcys(2012)/eGFRcr(2021); eGFRdiff, eGFRcys(2012) − eGFRcr(2021); TBMM, total body muscle mass; SD, standard deviation; CI, confidence interval; CHARLS, China Health and Retirement Longitudinal Study.
