# Supplementary material for: Comparative evaluation of the sarcopenia index and 5 related creatinine–cystatin C biomarkers in relation to all-cause mortality in older adults: A cohort study
Source: Medicine (Baltimore). 2026 Oct 2;105(40):e50842. doi: 10.1097/MD.0000000000050842 (PMC13632987; doi:10.1097/MD.0000000000050842)
Supplement: Supplementary file 6 [file medi-105-e50842-s006.pdf]

Supplementary Table S5. Incremental discrimination of six biomarkers added to the covariates-only baseline (Model 2), by sex in CHARLS 2015–2020

Comparison	Men				Women			
	N=2987; Death=428				N=2995; Death=228			
	Augmented C-index	ΔC-index (95% CI)	Augmented AUC_5y	ΔAUC_5y (95% CI)	Augmented C-index	ΔC-index (95% CI)	Augmented AUC_5y	ΔAUC_5y (95% CI)
Added SI	0.7331	0.0085 (0.0015, 0.0173)	0.7270	0.0095 (0.0008, 0.0210)	0.7640	0.0055 (-0.0009, 0.0175)	0.7571	-0.0022 (-0.0122, 0.0122)
Added TBMM	0.7310	0.0064 (0.0005, 0.0144)	0.7262	0.0087 (-0.0002, 0.0209)	0.7645	0.0061 (-0.0018, 0.0166)	0.7574	-0.0018 (-0.0142, 0.0150)
Added CCR	0.7301	0.0055 (0.0004, 0.0134)	0.7231	0.0056 (-0.0005, 0.0155)	0.7618	0.0033 (-0.0009, 0.0131)	0.7582	-0.0011 (-0.0087, 0.0086)
Added eGFRratio	0.7304	0.0058 (0.0005, 0.0136)	0.7243	0.0068 (-0.0000, 0.0169)	0.7602	0.0018 (-0.0013, 0.0106)	0.7574	-0.0019 (-0.0097, 0.0058)
Added eGFRdiff	0.7320	0.0074 (0.0015, 0.0161)	0.7248	0.0073 (-0.0002, 0.0178)	0.7610	0.0026 (-0.0008, 0.0100)	0.7572	-0.0020 (-0.0081, 0.0058)
Added pSMI	0.7297	0.0051 (-0.0000, 0.0124)	0.7249	0.0075 (-0.0006, 0.0179)	0.7616	0.0032 (-0.0033, 0.0118)	0.7592	-0.0001 (-0.0102, 0.0145)
Added 6	0.7370	0.0124	0.7327	0.0152	0.7693	0.0108	0.7637	0.0044 (-

biomarkers jointly	(0.0047, 0.0245)	(0.0036, 0.0327)	—	(0.0012, 0.0284)	0.0107, 0.0314)

Within sex, all comparisons used the same covariates-only baseline model; baseline C-index was 0.7246 in men and 0.7585 in women, and baseline AUC_5y was 0.7174 in men and 0.7593 in women. Paired event-stratified bootstrap resampling used B = 1000 for all comparisons. Positive Δ values indicate improved apparent discrimination after adding the biomarker(s) to the covariates-only baseline. AUC_5y, time-dependent area under the curve at 5 years.
