# Supplementary material for: Comparative evaluation of the sarcopenia index and 5 related creatinine–cystatin C biomarkers in relation to all-cause mortality in older adults: A cohort study
Source: Medicine (Baltimore). 2026 Oct 2;105(40):e50842. doi: 10.1097/MD.0000000000050842 (PMC13632987; doi:10.1097/MD.0000000000050842)
Supplement: Supplementary file 7 [file medi-105-e50842-s007.pdf]

Supplementary Table S6. Multicollinearity diagnostics (variance inflation factor and tolerance) for the six-biomarker joint model (Table 3), by sex in CHARLS 2015–2020

Biomarker	Men		Women	
	N=2,987; Death=428		N=2,995; Death=228	
	VIF	Tolerance	VIF	Tolerance
SI	32.4	0.031	37.9	0.026
pSMI	34.4	0.029	15.9	0.063
CCR	22.1	0.045	31.8	0.031
eGFRratio	20.9	0.048	24.5	0.041
eGFRdiff	9.9	0.101	8.8	0.114
TBMM	32.9	0.030	20.7	0.048

VIF and tolerance (1/VIF) were computed for the joint model in Table 3 (all six biomarkers entered simultaneously and adjusted for Model 2 covariates, as defined in Methods). Higher VIF (often >10) and lower tolerance indicate greater multicollinearity. SI, sarcopenia index (Cr × eGFRcys); pSMI, predicted skeletal muscle index; CCR, creatinine-to-cystatin C ratio; eGFRratio, eGFRcys(2012)/eGFRcr(2021); eGFRdiff, eGFRcys(2012) − eGFRcr(2021); TBMM, total body muscle mass; VIF, variance inflation factor; CHARLS, China Health and Retirement Longitudinal Study.
