# Supplementary material for: Comparative evaluation of the sarcopenia index and 5 related creatinine–cystatin C biomarkers in relation to all-cause mortality in older adults: A cohort study
Source: Medicine (Baltimore). 2026 Oct 2;105(40):e50842. doi: 10.1097/MD.0000000000050842 (PMC13632987; doi:10.1097/MD.0000000000050842)
Supplement: Supplementary file 8 [file medi-105-e50842-s008.pdf]

Supplementary Table S7. Supportive external analysis of sex-specific associations of six related creatinine-cystatin C biomarkers with all-cause mortality in NHANES 1999–2004, with an additional NHANES-derived ASM proxy (per 1-SD increase)

Biomarker	Men (N = 553)				Women (N = 624)			
	Death, N = 90				Death, N = 63			
	Unadjusted	Model 1	Model 2	Model 3†	Unadjusted	Model 1	Model 2	Model 3†
CCR	0.03 (0.00–0.22)**	0.06 (0.01–0.53)*	0.18 (0.03–1.07)	0.19 (0.03–1.20)	0.53 (0.40–0.72)***	0.61 (0.44–0.83)**	0.68 (0.46–0.99)*	0.69 (0.47–1.01)
pSMI	0.22 (0.08–0.61)**	0.37 (0.14–0.97)*	0.57 (0.22–1.47)	0.64 (0.23–1.82)	0.47 (0.32–0.69)***	0.56 (0.33–0.97)*	0.59 (0.38–0.92)*	0.55 (0.35–0.88)*
SI	0.34 (0.21–0.56)***	0.42 (0.24–0.74)**	0.60 (0.39–0.91)*	0.61 (0.39–0.95)*	0.49 (0.35–0.68)***	0.57 (0.41–0.80)**	0.66 (0.43–1.02)	0.68 (0.44–1.05)
eGFRratio	0.41 (0.25–0.66)***	0.48 (0.29–0.81)**	0.67 (0.46–0.98)*	0.68 (0.46–1.02)	0.62 (0.44–0.86)**	0.67 (0.49–0.93)*	0.75 (0.50–1.13)	0.78 (0.52–1.16)
eGFRdiff	0.41 (0.28–0.61)***	0.47 (0.30–0.75)**	0.61 (0.39–0.94)*	0.62 (0.40–0.98)*	0.56 (0.43–0.74)***	0.62 (0.46–0.83)**	0.69 (0.48–1.01)	0.71 (0.49–1.04)
TBMM	0.72 (0.47–1.12)	0.65 (0.45–0.96)*	0.81 (0.57–1.16)	0.87 (0.58–1.30)	0.59 (0.41–0.84)**	0.50 (0.33–0.77)**	0.54 (0.35–0.83)**	0.47 (0.30–0.76)**
Estimated ASM	0.86 (0.60–1.23)	0.92 (0.68–1.23)	1.04 (0.75–1.43)	1.22 (0.81–1.85)	0.68 (0.45–1.02)	0.66 (0.42–1.05)	0.67 (0.46–0.99)*	0.55 (0.34–0.88)*

Model 1 adjusted for age and waist-to-height ratio (WHtR). Model 2 additionally adjusted for poverty–income ratio (INDFMPIR), smoking, drinking, lung disease, hypertension, diabetes, cancer, stroke, psychiatric disease, liver disease, kidney disease, joint disease, log triglycerides (logTG), and log C-reactive protein (logCRP). † Model 3 additionally adjusted for DXA-derived low muscle mass (FNIH ALM/BMI) and was estimated in participants with available DXA (men N=548, deaths=90; women N=621, deaths=62). NHANES analyses used sex-stratified survey-weighted Cox models and were restricted to the locked intersection sample defined by complete data on the six primary biomarkers; the NHANES-derived ASM proxy (JCSM 2023 Eq.1) was additionally evaluated within this locked sample. NHANES, National Health and Nutrition Examination Survey; ASM, NHANES-derived estimated appendicular skeletal muscle mass; CCR, creatinine-to-cystatin C ratio; pSMI, predicted skeletal muscle index; SI, sarcopenia index (Cr × eGFRcys); eGFRratio, eGFRcys(2012)/eGFRcr(2021); eGFRdiff, eGFRcys(2012) − eGFRcr(2021); TBMM, total body muscle mass; WHtR, waist-to-height ratio.

*p<0.05, **p<0.01, ***p<0.001.
