# Supplementary material for: Comparative evaluation of the sarcopenia index and 5 related creatinine–cystatin C biomarkers in relation to all-cause mortality in older adults: A cohort study
Source: Medicine (Baltimore). 2026 Oct 2;105(40):e50842. doi: 10.1097/MD.0000000000050842 (PMC13632987; doi:10.1097/MD.0000000000050842)
Supplement: Supplementary file 9 [file medi-105-e50842-s009.pdf]

Supplementary Table S8. Supportive external analysis of mutually adjusted hazard ratios for all-cause mortality from a six-biomarker joint model, by sex in NHANES 1999–2004

Biomarker	Men			Women		
	N=553; Death=90			N=624; Death=63		
	HR (95% CI)	P	Best probability	HR (95% CI)	P	Best probability
SI	0.378 (0.103–1.385)	0.127	29.2%	0.464 (0.127–1.701)	0.233	29.5%
pSMI	2.308 (0.277–19.232)	0.422	5.5%	0.871 (0.167–4.536)	0.866	19.6%
CCR	0.586 (0.045–7.568)	0.671	36.8%	0.905 (0.254–3.221)	0.874	13.2%
eGFRratio	2.717 (0.706–10.450)	0.131	1.5%	2.298 (0.884–5.973)	0.079	0.1%
TBMM	0.680 (0.163–2.843)	0.583	11.6%	0.723 (0.105–4.961)	0.734	28.0%
eGFRdiff	0.701 (0.113–4.333)	0.691	15.4%	0.782 (0.307–1.994)	0.596	9.6%

Hazard ratios (HRs) are presented per 1-SD increase (z-score). Sex-stratified survey-weighted Cox models (svycoxph) included all six biomarkers simultaneously and were adjusted for Model 2 covariates. Analyses were restricted to the locked six-indicator intersection sample from NHANES 1999–2004 and used the WTSSCB6YR subsample weight. p values are two-sided Wald tests. Best probability reports bootstrap ranking stability (B = 1000), defined as the probability that a biomarker has the smallest hazard ratio (i.e., is most frequently ranked as the most protective) in the mutually adjusted model. In the present analyses, all biomarkers had NonNA = 1000; therefore, Best probability denominators did not differ across biomarkers. Best probability is intended as a stability-oriented comparative summary under severe collinearity rather than a direct measure of clinical utility. Given the limited number of deaths and strong intercorrelation among biomarkers in NHANES, these joint-model estimates should be interpreted as supportive directional evidence rather than definitive external comparative ranking. NHANES, National Health and Nutrition Examination Survey; CCR, creatinine-to-cystatin C ratio; pSMI, predicted skeletal muscle index; SI, sarcopenia index (Cr × eGFRcys); eGFRratio, eGFRcys(2012)/eGFRcr(2021); eGFRdiff, eGFRcys(2012) − eGFRcr(2021); TBMM, total body muscle mass.
