# Supplementary material for: Comparative evaluation of the sarcopenia index and 5 related creatinine–cystatin C biomarkers in relation to all-cause mortality in older adults: A cohort study
Source: Medicine (Baltimore). 2026 Oct 2;105(40):e50842. doi: 10.1097/MD.0000000000050842 (PMC13632987; doi:10.1097/MD.0000000000050842)
Supplement: Supplementary file 10 [file medi-105-e50842-s010.pdf]

Supplementary Table S9. Supportive external analysis of pairwise comparisons of joint-model coefficients among six biomarkers, by sex in NHANES 1999–2004

Comparison	Men			Women		
	N=553; Death=90			N=624; Death=63		
	Diff (βA – βB)	q(BH)	Pr(βA < βB)	Diff (βA – βB)	q(BH)	Pr(βA < βB)
SI vs pSMI	-1.810	0.667	82.8%	-0.630	0.967	64.7%
SI vs CCR	-0.439	0.983	54.3%	-0.668	0.967	67.1%
SI vs eGFRratio	-1.973	0.667	85.9%	-1.599	0.650	94.0%
SI vs TBMM	-0.588	0.908	65.0%	-0.443	0.967	60.4%
SI vs eGFRdiff	-0.618	0.908	66.8%	-0.522	0.967	67.2%
pSMI vs CCR	1.371	0.908	25.7%	-0.038	0.967	49.1%
pSMI vs eGFRratio	-0.163	0.983	55.4%	-0.970	0.967	84.5%
pSMI vs TBMM	1.222	0.908	31.5%	0.187	0.967	50.8%
pSMI vs eGFRdiff	1.192	0.667	18.2%	0.108	0.967	47.5%
CCR vs eGFRratio	-1.534	0.778	82.8%	-0.932	0.967	86.6%
CCR vs TBMM	-0.149	0.983	60.6%	0.225	0.967	50.2%
CCR vs eGFRdiff	-0.179	0.983	56.0%	0.146	0.967	47.8%
eGFRratio vs TBMM	1.385	0.667	14.5%	1.156	0.967	22.0%
eGFRratio vs eGFRdiff	1.355	0.832	19.7%	1.078	0.960	8.1%
TBMM vs eGFRdiff	-0.030	0.983	50.1%	-0.079	0.967	47.0%

Diff (βA − βB) is the difference in standardized (z-score) coefficients between biomarker A and biomarker B from the mutually adjusted six-biomarker joint Cox model (sex-stratified; additionally adjusted for Model 2 covariates as defined in the main-text Methods). q(BH) denotes Benjamini-Hochberg FDR-adjusted p-values for the 15 within-sex pairwise contrasts. Pr(βA < βB) is the bootstrap-estimated probability that βA is more protective (smaller coefficient) than βB, based on the joint-model bootstrap (B = 1000). Abbreviations: SI, sarcopenia index (Cr × eGFRcys); pSMI, predicted skeletal muscle index; CCR, creatinine-to-cystatin C ratio; eGFRratio, eGFRcys(2012)/eGFRcr(2021); eGFRdiff, eGFRcys(2012) − eGFRcr(2021); TBMM, total body muscle mass; SD, standard deviation; CI, confidence interval; NHANES, National Health and Nutrition Examination Survey.
