# Supplementary material for: Comparative evaluation of the sarcopenia index and 5 related creatinine–cystatin C biomarkers in relation to all-cause mortality in older adults: A cohort study
Source: Medicine (Baltimore). 2026 Oct 2;105(40):e50842. doi: 10.1097/MD.0000000000050842 (PMC13632987; doi:10.1097/MD.0000000000050842)
Supplement: Supplementary file 11 [file medi-105-e50842-s011.pdf]

Supplementary Table S10. IPW sensitivity analyses for missing-data-related inclusion in CHARLS 2015–2020

Panel A. Single-biomarker Model 2 sensitivity			
Biomarker	Contrast	Men	Women
SI	Unweighted	0.771 (0.676–0.878)	0.789 (0.649–0.959)
SI	IPW	0.771 (0.657–0.905)	0.776 (0.600–1.004)
TBMM	Unweighted	0.722 (0.630–0.827)	0.679 (0.572–0.807)
TBMM	IPW	0.708 (0.614–0.815)	0.672 (0.552–0.818)
pSMI	Unweighted	0.737 (0.639–0.851)	0.690 (0.568–0.837)
pSMI	IPW	0.725 (0.623–0.842)	0.676 (0.542–0.844)
CCR	Unweighted	0.831 (0.735–0.940)	0.852 (0.712–1.020)
CCR	IPW	0.833 (0.719–0.966)	0.844 (0.679–1.048)
eGFRratio	Unweighted	0.815 (0.717–0.925)	0.881 (0.741–1.048)
eGFRratio	IPW	0.817 (0.698–0.957)	0.869 (0.702–1.077)
eGFRdiff	Unweighted	0.805 (0.724–0.895)	0.875 (0.759–1.008)
eGFRdiff	IPW	0.806 (0.717–0.907)	0.870 (0.749–1.011)

Panel B. Joint-model ranking sensitivity			
Biomarker	Contrast	Men	Women
SI	Original	0.875	0.947
SI	IPW	0.862	0.964

Panel B. Joint-model ranking sensitivity			
Biomarker	Contrast	Men	Women
TBMM	Original	0.085	0.047
TBMM	IPW	0.111	0.029
pSMI	Original	0.021	0.003
pSMI	IPW	0.013	0.004
CCR	Original	0.000	0.001
CCR	IPW	0.000	0.000
eGFRratio	Original	0.000	0.001
eGFRratio	IPW	0.003	0.003
eGFRdiff	Original	0.019	0.001
eGFRdiff	IPW	0.011	0.000

Panel A reports sex-specific per 1-SD hazard ratios from single-biomarker Cox models under the original complete-case analysis ("Unweighted") and after inverse probability weighting for inclusion ("IPW"). Panel B reports Best probability from the mutually adjusted six-biomarker joint model under the original analysis ("Original") and after IPW ("IPW"). In Panel B, Best probability is defined as the proportion of bootstrap resamples (B = 1000) in which a biomarker had the most protective standardized coefficient in the joint model. Model 2 covariates were defined as in the main-text Methods. IPW, inverse probability weighting; SI, sarcopenia index (Cr × eGFRcys); pSMI, predicted skeletal muscle index; CCR, creatinine-to-cystatin C ratio; eGFRratio, eGFRcys(2012)/eGFRcr(2021); eGFRdiff, eGFRcys(2012) − eGFRcr(2021); TBMM, total body muscle mass; CHARLS, China Health and Retirement Longitudinal Study.
